# Heart failure with a preserved ejection fraction: synergy between cardiac and extracardiac mechanisms and effects of novel therapies

**DOI:** 10.1007/s10741-026-10671-x

**Published:** 2026-09-21

**Authors:** Soufiane Nassiri, M. Louis Handoko, Arno A. van de Bovenkamp, Vanessa P.M. van Empel, Daniël H. van Raalte, Etto C. Eringa

**Affiliations:** 1 Departments of Cardiology, Diabetes Centre, Internal Medicine Amsterdam, Physiology, University Medical Centre, Amsterdam Cardiovascular Sciences, De Boelelaan 1108, 1081 HZ Amsterdam, The Netherlands; 2 Amsterdam Cardiovascular Sciences, Amsterdam, The Netherlands; 3 Department of Cardiology, Transplantation Centre, University Medical Centre Utrecht, Utrecht, The Netherlands; 4 Department of Cardiology, Physiology, Cardiovascular Research Institute Maastricht (CARIM), Maastricht University, Maastricht, the Netherlands

**Keywords:** HFpEF, Obesity, Type 2 diabetes, Chronic kidney disease, Microvascular dysfunction, Cardio-kidney metabolic syndrome, Sodium-glucose cotransporter 2 inhibitors, Glucagon-like peptide-1 receptor agonists, Mineralocorticoid receptor antagonists

## Abstract

Heart failure with preserved ejection fraction (HFpEF) is a systemic disease in which metabolic comorbidities obesity, type 2 diabetes (T2D), and chronic kidney disease (CKD) converge to drive systemic inflammation, endothelial dysfunction, and myocardial fibrosis. Microvascular dysfunction represents an important link between these comorbidities and abnormalities within and beyond the heart, although its causal contribution to HFpEF remains incompletely established. In addition to promoting myocardial stiffness and diastolic dysfunction, impaired microvascular function across the pulmonary and peripheral circulations may contribute to reduced tissue perfusion, exercise intolerance, and dyspnoea. CKD may further aggravate this process through volume and neurohormonal pathways as well as circulating uraemic and inflammatory factors that impair endothelial function, through a kidney–microvascular axis. Despite its high prevalence and poor prognosis, therapeutic options for HFpEF have historically been limited. Recent advances, including sodium–glucose cotransporter 2 inhibitors (SGLT2i), glucagon-like peptide-1 (GLP-1) receptor agonists and dual incretin therapies (GLP-1/GIP RAs), and (non-steroidal) mineralocorticoid receptor antagonists, have reshaped the therapeutic landscape by improving cardiovascular and renal outcomes in patients with HFpEF. These agents exert complementary effects on hemodynamic stress, metabolic dysregulation, inflammation, and fibrosis, and may also improve microvascular function across multiple vascular beds. In this narrative review, we provide an organ-by-organ synthesis of the pathophysiological interactions among the heart, kidney, systemic microvasculature, pulmonary circulation, and skeletal muscle in CKMS-related HFpEF. We further map the clinical and mechanistic effects of aforementioned therapies across these interconnected organ systems and highlight areas in which mechanistic and clinical evidence gaps remain.

**Graphical abstract:**

**Figure:**
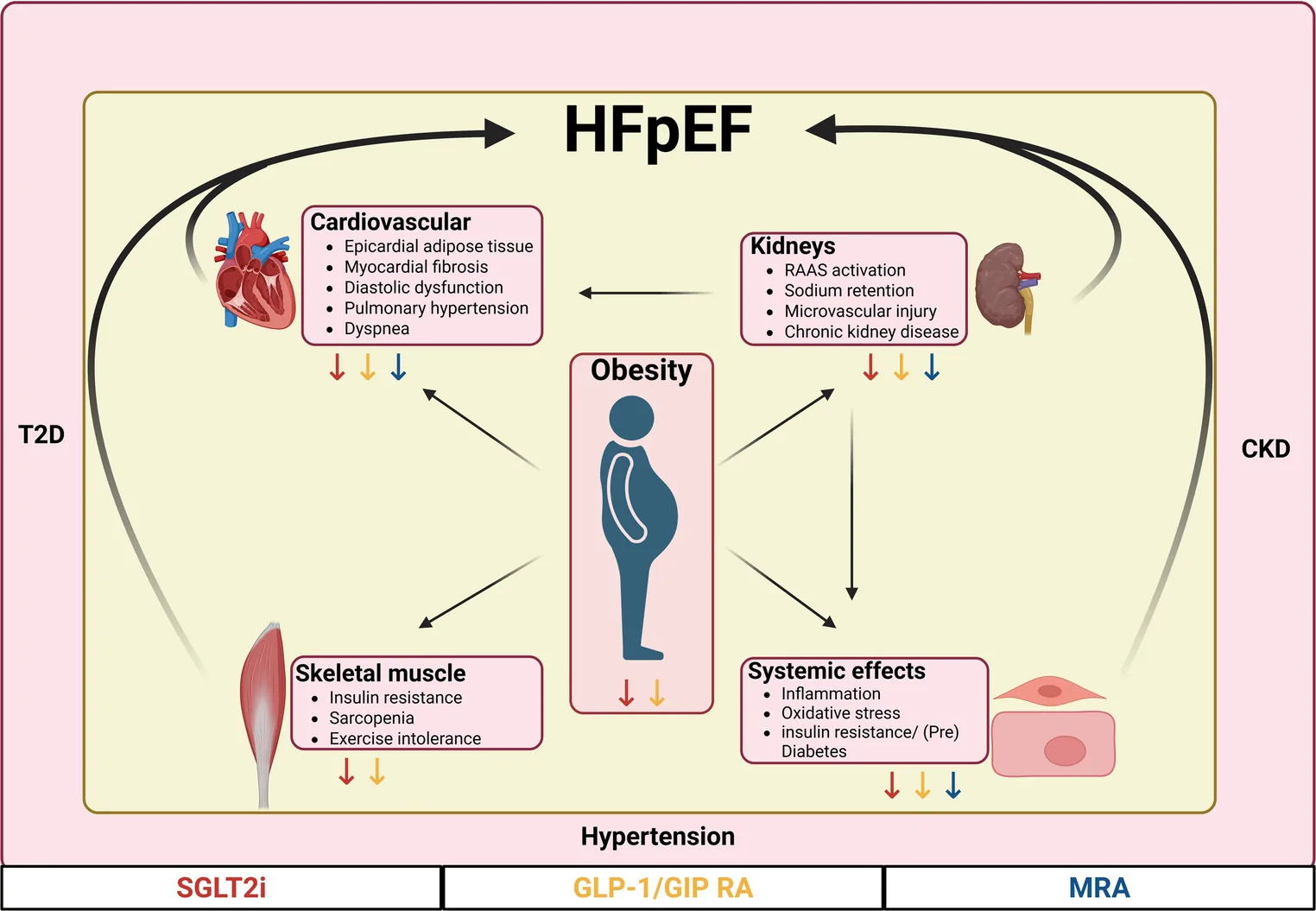

## Research insights

**What is currently known about this topic?:**

- HFpEF is a systemic disease involving heart, kidneys, lungs, skeletal muscle and microvasculature.
- CKMS denotes the interconnected dysfunction of the cardiovascular, renal, and metabolic systems; This review highlights the relation between HFpEF, CKD and obesity/metabolic dysregulation.
- Recent agents including SGLT2i, GLP-1/GIP RAs, and MRAs have demonstrated clinically meaningful improvements in cardiovascular and renal outcomes.

**What is the key research question?:**

What are the pathophysiological links between obesity, T2D, CKD, and HFpEF within the context of the CKMS, and how are they affected by novel therapies?

**What is new?:**

- This review provides an organ-by-organ synthesis of CKMS-related HFpEF, integrating pathophysiological mechanisms across the heart, kidney, systemic microvasculature, pulmonary circulation, and skeletal muscle.
- Microvascular dysfunction is considered as a potential cross-organ interface linking metabolic and kidney dysfunction to cardiac and extracardiac manifestations of HFpEF, while its causal contribution remains to be fully characterised.
- The clinical outcomes and mechanistic effects of SGLT2i, GLP-1/GIP RAs, and MRAs are mapped across these interconnected organ systems, highlighting complementary therapeutic effects and remaining evidence gaps.

**How might this study influence clinical practice?:**

This review describes a mechanistic rationale for multi-organ treatment strategies for HFpEF in the context of CKMS.

## Introduction

The lifetime risk of heart failure (HF) is steadily rising up to 24%, with women at greater risk of heart failure with preserved ejection fraction (HFpEF) [1]. According to the European Society of Cardiology, HFpEF is defined by the presence of typical HF symptoms such as dyspnoea and/or exercise intolerance combined with a left ventricular ejection fraction of ≥ 50% and evidence of diastolic dysfunction and/or elevated left ventricular filling pressures [2]. HFpEF is a complex, heterogeneous, multisystem syndrome involving the heart, kidneys, lungs, adipose tissue, and skeletal muscle. It is commonly associated with obesity, type 2 diabetes (T2D), hypertension, chronic kidney disease (CKD), microvascular dysfunction, and dyslipidaemia. This clustering of comorbidities highlights the need for treatment strategies that target the multifactorial nature of HFpEF, particularly the interplay between cardiovascular, kidney, and metabolic dysfunction.

This paradigm is reflected in the cardio-kidney metabolic syndrome (CKMS) framework, proposed as an overarching construct that integrates the continuum of cardiovascular, kidney, and metabolic disease. Aging, hypertension, T2D, obesity, and kidney dysfunction promote chronic systemic inflammation and microvascular dysfunction [3]. A hallmark of this process is endothelial dysfunction, largely due to impaired nitric oxide (NO) bioavailability, which can contribute to cardiomyocyte stiffness, hypertrophy, and myocardial fibrosis, key drivers of left ventricular diastolic dysfunction and elevated filling pressures [4]. These processes elevate filling pressures and result in the cardiac symptoms of HFpEF. The aforementioned pressures have also been demonstrated to exert a stress effect on the lungs, manifesting as dyspnoea [5]. This pulmonary congestion is aggravated by reduced skeletal muscle perfusion and capillary density which result in fatigue, muscle atrophy, and diminished exercise capacity [3]. 

While therapeutic advances have improved outcomes in heart failure with reduced ejection fraction (HFrEF), most remain ineffective in HFpEF. Recent therapies, including sodium–glucose cotransporter 2 inhibitors (SGLT2i), glucagon-like peptide-1 (GLP-1) and GLP-1/Glucose-dependent insulinotropic polypeptid receptor agonists (GIP RAs), and nonsteroidal mineralocorticoid receptor antagonists (e.g., finerenone), reduce HF events and cardiovascular death in HFpEF [6–8]. Notably, dual GLP-1/GIP RAs appear to confer additional metabolic and adipose-tissue benefits beyond GLP-1 receptor agonism alone, which may be particularly relevant in the pathophysiology of HFpEF [6]. Despite these advances, residual risk remains high, underscoring the need for deeper mechanistic insight to identify novel therapeutic targets.

Given the insights summarized above, HFpEF is increasingly viewed within the broader cardio-kidney-metabolic syndrome (CKMS), an overarching umbrella construct that integrates cardiovascular, kidney, and metabolic disease, rather than as a isolated cardiac disorder [9]. Several components of this pathophysiological framework have been reviewed separately. These include the bidirectional relationship between CKD and HFpEF, the pleiotropic cardiovascular and kidney effects of GLP-1 receptor agonists, and the role of systemic microvascular dysfunction in HFpEF. However, these mechanisms are often considered in isolation. 

In this narrative review we aim to integrate the pathophysiological interactions between obesity, T2D, CKD, and HFpEF within the CKMS framework through an organ-by-organ synthesis of the heart, kidney, microvasculature, pulmonary circulation, and skeletal muscle. We specifically examine microvascular dysfunction as a mediator of inter-organ crosstalk, including the kidney–microvasculature axis, and map the mechanistic and clinical effects of SGLT2i, GLP-1/GIP RAs, and MRAs across these organ systems. Finally, we identify areas in which mechanistic or clinical evidence gaps remain.

### Pathophysiology of the cardio-kidney-metabolic syndrome

CKMS denotes the interconnected dysfunction of the cardiovascular, kidney, and metabolic systems; highlighting the associations between HFpEF, CKD and metabolic dysregulation, most notably insulin resistance. The different aspects of metabolic dysregulation in CKMS are collectively termed as metabolic syndrome [9]. In this review, we further emphasize the role of excessive adipose tissue and T2D in this syndrome.

### Obesity as a substrate for HFpEF and metabolic syndrome

Obesity is a central driver of the metabolic syndrome and has profound effects on cardiovascular outcomes in HF, as evidenced by an increased risk of all-cause mortality and HF hospitalizations and contributes to HFpEF through multiple interconnected pathways, including systemic inflammation, neurohormonal activation, and metabolic dysregulation, all of which impair cardiovascular structure and function [10, 11]. The presence of pericardial restraint and systolic left ventricular eccentric remodelling leads to ventricular interdependence and reliance on plasma volume expansion [12]. 

Excess total and visceral adipose tissue promotes the secretion of adipokines, which stimulate inflammatory pathways and chronic low-grade inflammation [13]. Within the myocardium, the processes outlined above induce cardiomyocyte remodelling by means of endothelial dysfunction and increased cardiomyocyte stiffness and fibrosis, thereby facilitating the development and progression of diastolic dysfunction in HFpEF [14]. Hemodynamically, obesity increases total venous return to the ventricles and increases cardiac filling pressures. Chronic elevation of filling pressures drives atrial and ventricular remodelling, aggravates diastolic dysfunction, and promotes pulmonary hypertension [15]. 

The kidneys are similarly vulnerable to the adverse effects of obesity. Visceral adiposity and type 2 diabetes are strongly associated with CKD progression, mediated through Renin-angiotensin-aldosterone system (RAAS) activation, oxidative stress, capillary rarefaction, stiffening of large arteries, and sodium retention affecting pulse pressure, afterload and vital organ blood flow [16, 17]. Perinephric fat including kidney sinus fat also exerts paracrine and endocrine effects through the secretion of cytokines, adipokines, and metabolites, which may disrupt kidney vascular and glomerular function (by inducing glomerular hyperfiltration), and aggravate microvascular injury in the myocardium, accelerating kidney and endothelial impairment [18, 19]. 

Beyond the heart and kidneys, obesity exerts systemic effects that aggravate HFpEF. Increased (abdominal) fat is associated with decreased insulin sensitivity [20]. In skeletal muscle, obesity-induced systemic pro-inflammatory milieu and impaired energy metabolism further compromise oxygen delivery and utilization in muscle tissue, worsening exercise intolerance [21]. 

### Role of type 2 diabetes in HFpEF

T2D is strongly linked to adverse cardiovascular outcomes. Kidney disease associated with hyperglycaemia, known as diabetic kidney disease (DKD), is closely associated with excess adiposity [22]. While hyperglycaemia contributes to cardiovascular risk in T2D, adiposity-driven metabolic disturbances appear more central to the development of HFpEF. In support of an indirect relationship of hyperglycaemia with HFpEF, the EMPEROR-PRESERVED trial demonstrated that SGLT2i reduce the risk of cardiovascular outcomes regardless of diabetes status, highlighting the importance of glucose-independent mechanisms in HFpEF pathophysiology [23]. Metabolic disturbances in prediabetes and T2D include hyperglycaemia, hyperlipidaemia, and accumulation of advanced glycation end products (AGEs). These abnormalities promote endothelial dysfunction, microvascular rarefaction, myocardial fibrosis, and impairment of diastolic function [24]. This increases ventricular stiffness and left ventricular filling pressures, and contributing to pulmonary hypertension.

Beyond the heart, hyperglycaemia and insulin resistance are related to systemic changes in adipose tissue, kidneys, and skeletal muscle. In the kidneys, chronic hyperglycaemia and activation of the RAAS system impairs glomerular and tubular function, exacerbating sodium retention, congestion, and fluid overload [25]. In skeletal muscle, insulin resistance and reduced microvascular perfusion contribute to mitochondrial dysfunction, sarcopenia, and exercise intolerance [26, 27]. Importantly, excess adiposity remains the pivotal upstream driver that fuels insulin resistance and systemic inflammation, establishing the metabolic environment in which HFpEF develops and progresses in both diabetic and non-diabetic individuals [28]. 

### Chronic kidney disease and HFpEF

Impaired kidney function is frequently observed in patients with HFpEF. Within the metabolic syndrome, insulin resistance, adipose tissue dysfunction, inflammation, oxidative stress, endothelial dysfunction, and activation of the RAAS all contribute to kidney damage.

Kidney injury often manifests as albuminuria, glomerular sclerosis, and enhanced sodium reabsorption, processes further aggravated by dysregulated levels of adipokines such as leptin [29]. Ectopic fat deposition and chronic low-grade inflammation accelerate glomerular hypertrophy and interstitial fibrosis, reducing kidney functional reserve [30]. In parallel, increased aldosterone concentrations are often observed, which exacerbates kidney vascular injury, fibrosis, and fluid retention [31], while hyperglycaemia-driven AGE accumulation directly damage endothelial cells and accelerates CKD progression [32]. 

HFpEF and CKD share interrelated pathophysiological mechanisms, whereby impaired kidney function promotes fluid overload, systemic inflammation, and neurohormonal activation, contributing to cardiac stiffness and diastolic dysfunction. Beyond these mechanisms, kidney dysfunction also contributes through a humoral kidney–microvascular signaling axis with known (RAAS) and unknown mediators. Accumulation of uraemic toxins in CKD promotes endothelial inflammation and oxidative stress through pathways including reactive oxygen species (ROS) and nuclear factor κB (NF-κB) signalling, impairing endothelial function [33]. 

### Systematic oxidative stress and inflammation in HFpEF: role of the microvasculature

**Fig. 1:**
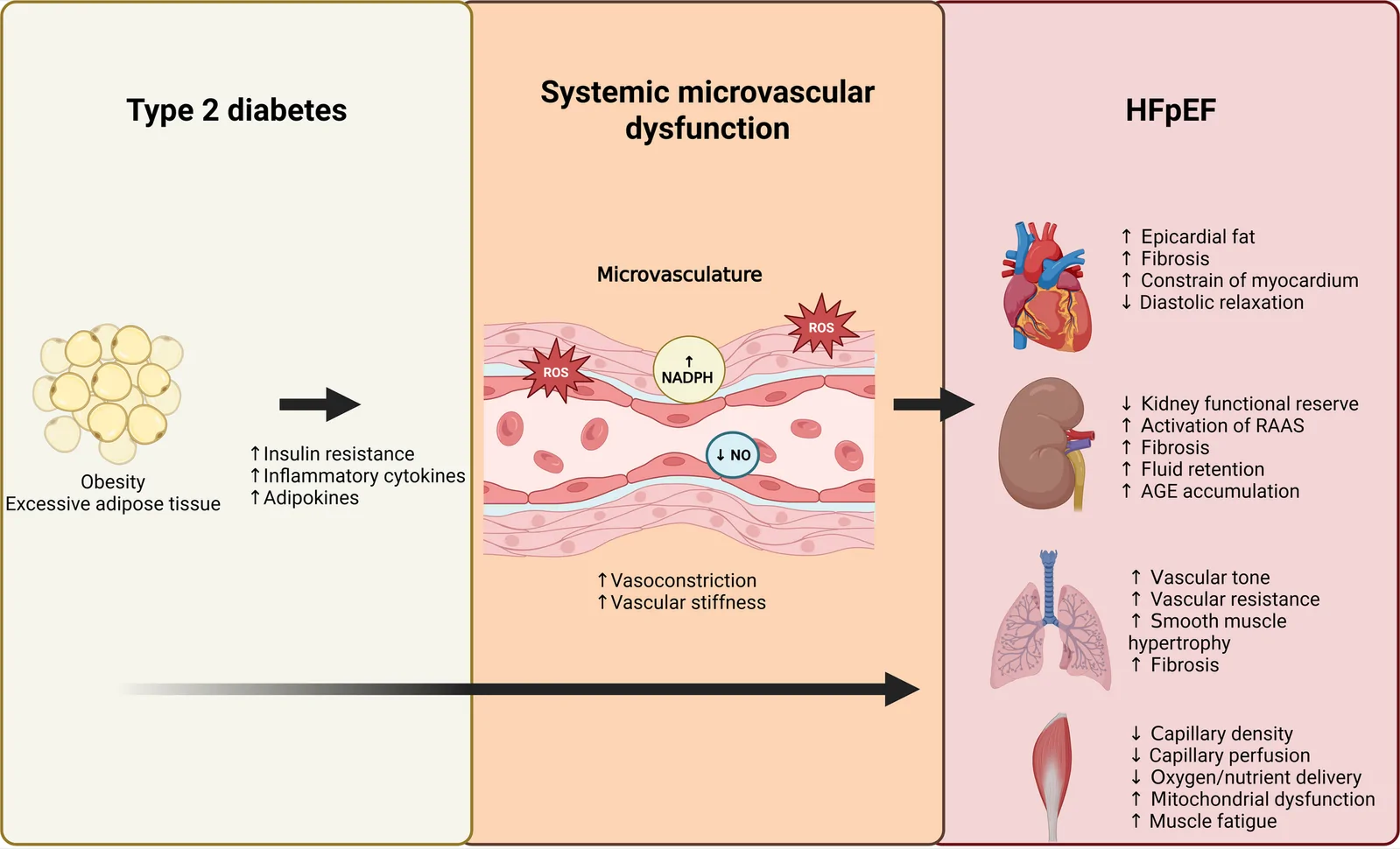
Role of the metabolic syndrome and its effect on systemic microvascular dysfunction and HFpEF. Reactive oxygen species (ROS); nicotinamide adenine dinucleotide phosphate (NADPH); Advanced glycation end products (AGE); renin-angiotensin-aldosterone system (RAAS) Created in BioRender.com

Systemic oxidative stress and inflammation in obesity, T2D and CKD drive microvascular dysfunction across the heart and extra-cardiac organs (Fig. 1), with insulin resistance as an important contributor [34]. Under physiological conditions, insulin promotes NO production and vascular smooth muscle relaxation through activation of the phosphatidylinositol 3-kinase (PI3K)/Akt pathway, which phosphorylates endothelial nitric oxide synthase (eNOS) [35]. In CKMS, pro-inflammatory cytokines (e.g., TNF-α, IL-6, MCP-1), endothelin-1, and adipokines promote oxidative stress and endothelial dysfunction through activating NADPH oxidase enzymes and ROS production [36, 37]. ROS reduce NO bioavailability and impair vasodilation, resulting in increased vascular tone and stiffness [38]. CKD may further contribute to this process through circulating uraemic factors that promote endothelial inflammation and oxidative stress [33]. 

In the heart, adipose tissue expansion, particularly of epicardial fat, enhances secretion of pro-inflammatory adipokines and chemokines, fuelling chronic myocardial inflammation, immune cell recruitment, and interstitial fibrosis [11, 14]. It has been demonstrated that endothelial dysfunction, amplified by oxidative stress, inflammation, and insulin resistance, impairs NO-mediated vasodilation, increasing myocardial stiffness and impairs diastolic relaxation [14]. Furthermore, the physical presence of excessive epicardial fat may mechanically constrain the myocardium, restricting filling and further worsening diastolic function [12]. 

In skeletal muscle, endothelial dysfunction reduces capillary density and perfusion, thereby limiting oxygen and nutrient delivery during exercise [39]. Mitochondrial dysfunction, exacerbated by excess fatty acid exposure, drives oxidative stress and impairs muscle energetics [39]. Along with microvascular rarefaction and impaired NO signalling, these abnormalities contribute to a mismatch between metabolic demand and supply, decreased muscle strength and exercise intolerance in patients with HFpEF. Chronic exposure to inflammatory cytokines and hyperglycaemia further aggravates endothelial injury and ROS generation, amplifying local dysfunction in the muscles.

Given that dyspnoea and exercise intolerance are hallmark symptoms of HFpEF, pulmonary involvement plays a central role in symptom development. In the lungs, systemic and local microvascular inflammation induce pulmonary endothelial dysfunction as a consequence of an imbalance between NO and endothelin-1 secretion, increasing vascular tone and resistance [40]. Over time, pulmonary vascular remodelling, including smooth muscle hypertrophy and fibrosis, raises pulmonary capillary pressures and contributes to pulmonary hypertension [41]. Concurrently, chest wall and visceral adiposity mechanically restrict ventilation, reduce lung compliance, while inflammatory mediators further aggravate endothelial injury within the pulmonary vasculature [40]. 

Although these observations support a role for microvascular dysfunction in HFpEF, its precise causal contribution remains incompletely known. Much of the available human evidence is cross-sectional or associative, making it difficult to distinguish whether peripheral microvascular dysfunction contributes directly to HFpEF pathophysiology or represents a parallel manifestation of shared upstream comorbidities, including obesity, T2D, CKD, ageing, and systemic inflammation.

### Mechanistic effects of novel therapies

**Fig. 2:**
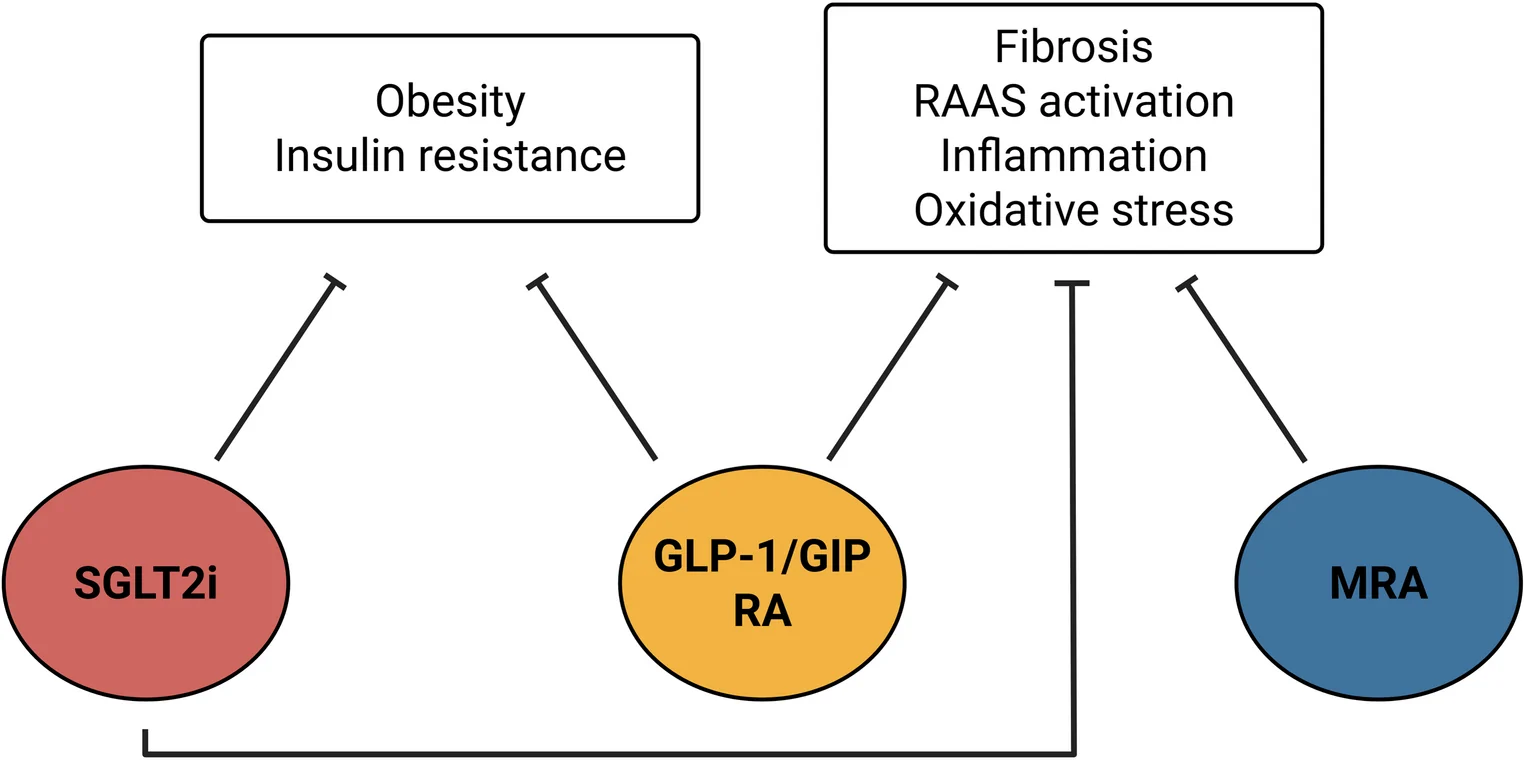
Pleiotropic and systemic effects of SGLT2i, GLP-1/GIP RA, and MRA

In the previous section, we have summarized the pathophysiological mechanisms in and outside of the heart contributing to HFpEF. In the past decade, novel therapies have demonstrated clinical improvements in HFpEF with relevant effects on important (metabolic) comorbidities. In the following section, we will focus on the clinical effects of these novel therapies and how they impact mechanisms contributing to HFpEF (Fig. 2).

### SGLT2i in HFpEF and CKD

**Fig. 3:**
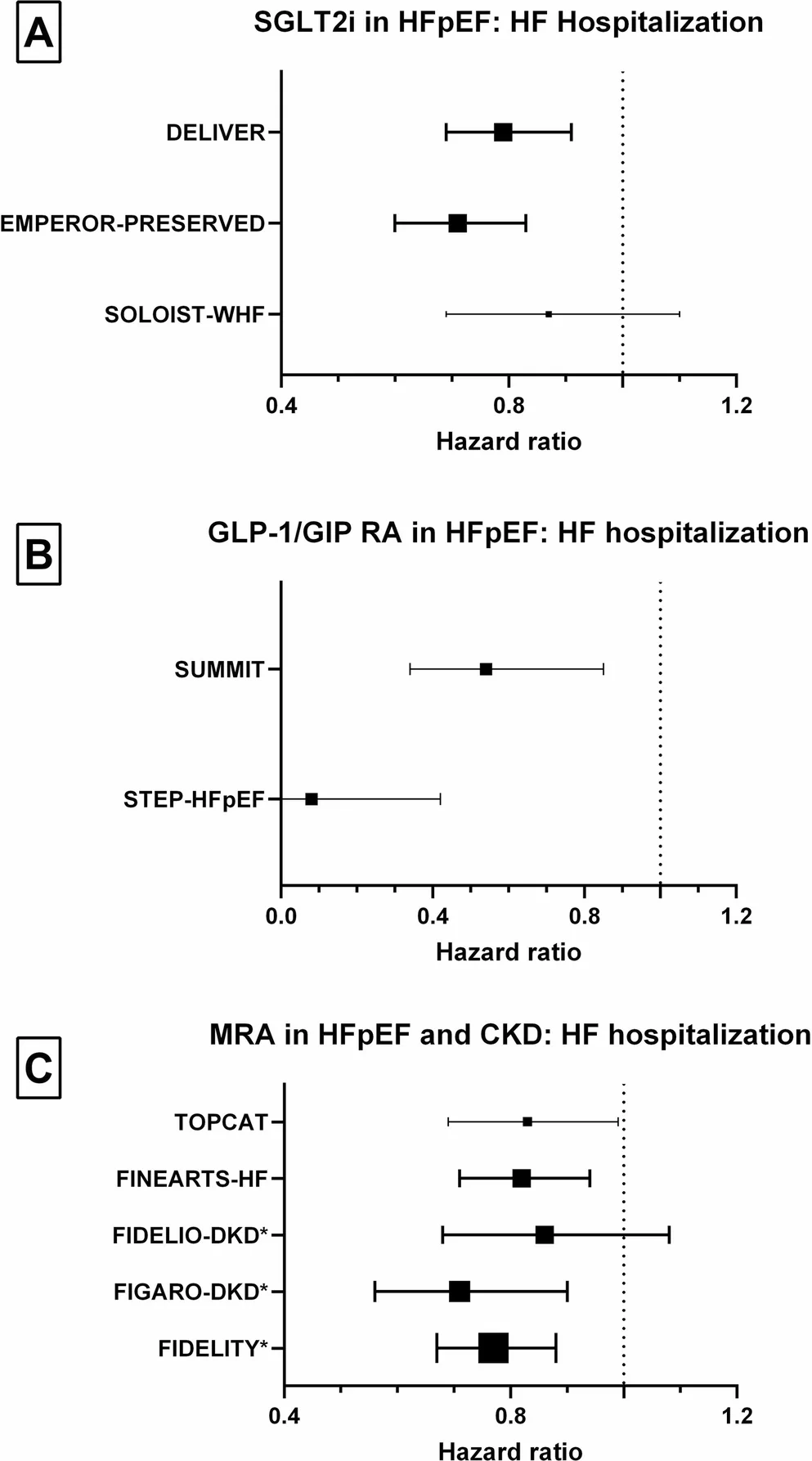
Forest plot demonstrating the effect of SGLT2i, GLP-1/GIP RA, and MRAs on hospitalization for heart failure. (**A**) HFpEF (SGLT2i); (**B**) HFpEF (GLP-1/GIP RA); (**C**) HFpEF/CKD (MRAs). *HF patients were not stratified for HFpEF or HFrEF

Recent trials have demonstrated the efficacy of SGLT2i in patients with HFpEF. In DELIVER, EMPEROR-PRESERVED, and SOLOIST-WHF relative risk reductions (RRR) of 21% to 34% were observed for the composite endpoint of cardiovascular death or worsening HF, defined as hospitalization or urgent visits for HF (Table 1) [8, 23, 42]. Specifically, when examining hospitalization for heart failure, these trials demonstrated a RRR of 21% to 29%, with the subgroup analysis of LVEF ≥ 50% in the SOLOIST-WHF trial taken into consideration (Fig. 3). It is important to note that reduction in the composite endpoint was mainly attributed to the reduction in HF hospitalizations and not to a reduction in cardiovascular mortality. Further subgroup analysis for the composite primary outcome in patients with HFpEF and CKD experienced RRR of 19% and 22% [8, 23]. In HFpEF with obesity (BMI ≥ 30 kg/m²), RRR for composite endpoint of cardiovascular death or worsening HF ranging from 15% to 26% [8, 23]. These benefits were consistent irrespective of diabetes status. In patients with diabetes, RRR for HF outcomes ranged from 17% to 21% in HFpEF [8, 23]. Comparable reductions were observed in non-diabetic HFpEF patients, with RRR 19% to 22% in [8, 23].

**Table 1:** Effects of SGLT2i in HFpEF including subgroup analysis of CKD, obesity, and T2D

Trial	Population	Primary endpoint	Primary outcome HR (95%CI)	Subgroups HR (95% CI)
DELIVER [8] Dapagliflozin vs. placebo	*N* = 6263; HFpEF/HFmrEF; elevated NT-proBNP; NYHA II-IV; ambulatory or hospitalized	Composite of worsening heart failure or CV death	Primary endpoint: 0.82 (0.73–0.92) HHF: 0.79 (0.69–0.91) CV death: 0.88 (0.74–1.05)	LVEF 50–59%: 0.79 (0.65–0.97) LVEF ≥ 60%: 0.78 (0.62–0.98) eGFR < 60: 0.81 (0.69–0.94) BMI ≥ 30: 0.74 (0.63–0.88) Diabetes: 0.83 (0.70–0.97) No diabetes: 0.81 (0.68–0.96)
EMPEROR-PRESERVED [23] Empagliflozin vs. placebo	*N* = 5988; HFpEF/HFmrEF; elevated NT-proBNP; NYHA II-IV; ambulatory or hospitalized	Composite of worsening heart failure or CV death	Primary endpoint: 0.79 (0.69–0.90) HHF: 0.71 (0.60–0.83) CV death: 0.92 (0.76–1.09)	LVEF 50–59: 0.80 (0.64–0.99) LVEF ≥ 60 0.87 (0.69–1.10) eGFR < 60 0.78 (0.66–0.91) BMI ≥ 30: 0.85 (0.70–1.03) Diabetes: 0.79 (0.67–0.94) No diabetes: 0.78 (0.64–0.95)
SOLOIST-WHF [42] Sotagliflozin vs. placebo	*N* = 1222; T2D with recent decompensated HF and elevated natriuretic peptides	Composite of worsening heart failure or CV death	Primary endpoint: 0.67 (0.52–0.85)	LVEF ≥ 50: 0.66 (0.38–1.15) eGFR < 60: 0.59 (0.44–0.79) eGFR ≥ 60: 0.90 (0.58–1.37) BMI ≥ 30: 0.61(0.44–0.86)

Effect of SGLT2i in Chronic Kidney Disease. Body mass index (BMI); Confidence interval (CI); Chronic kidney disease (CKD); Cardiovascular (CV); Estimated glomerular filtration rate (eGFR); Heart failure (HF); Hospitalization for heart failure (HHF); Hazard ratio (HR); Left ventricular ejection fraction (LVEF); N-terminal pro–B-type natriuretic peptide (NT-proBNP); New York Heart Association (NYHA); Sodium-glucose cotransporter-2 inhibitor (SGLT2i); Type 2 diabetes mellitus (T2DM)

In various clinical trials involving patients with CKD, SGLT2i demonstrated substantial RRR for composite endpoints that included reduction of kidney failure, cardiovascular death, and hospitalization for HF, with reported reductions ranging from 26% to 39% [43–46]. Focusing specifically on kidney disease progression, defined by outcomes such as a sustained decline in estimated glomerular filtration rate (eGFR), kidney failure or kidney death, RRRs of 34% to 44% were observed [43, 46]. With regard to hospitalization for heart failure SGLT2i were consistently associated with meaningful risk reductions. Across trials, RRRs of 16% to 33% were reported for worsening HF events and cardiovascular death for patients with CKD (Table 2) [43–45]. Notably, only the SCORED trial performed a dedicated subgroup analysis in patients with HF-related criteria and a LVEF ≥ 50%, demonstrating a 42% RRR for the composite endpoint, which included hospitalization for heart failure. Apart from SCORED, no trials provided stratified analyses distinguishing HFpEF from HFrEF populations.

**Table 2:** Effect of SGLT2i in CKD including subgroup analysis of heart failure, obesity, and T2D

Trial	Population	Primary endpoint	Primary outcome HR (95%CI)	Subgroups HR (95% CI)
DAPA-CKD [43] Dapagliflozin vs. placebo	*N* = 4304; CKD (eGFR 25–75, uACR 200–5000) on RAAS blockade	Composite of ≥ 50% eGFR decline, ESKD, or renal/CV death or HF hospitalization	Primary endpoint: 0.61 (0.51–0.72) WRF: 0.56 (0.45–0.68) CV death or HHF: 0.71 (0.55–0.92)	eGFR < 45: 0.63 (0.51–0.78) eGFR ≥ 45: 0.49 (0.34–0.69) Diabetes: 0.64 (0.52–0.79) No diabetes: 0.50 (0.35–0.72)
EMPA-KIDNEY [44] Empagliflozin vs. placebo	*N* = 6609; CKD (eGFR 20–90 with albuminuria > 200 mg/g at screening); on RAAS blockade	Composite of progression of kidney disease (ESKD, eGFR < 10 ml/min/1.73m2, ≥ 40% eGFR decline, death from renal causes) or death from CV causes	Primary endpoint: 0.72 (0.64–0.82) Death from CV causes or HHF: 0.84 (0.67–1.07)	eGFR < 30: 0.73 (0.62–0.86) eGFR ≥ 30 to < 45: 0.78 (0.62–0.97) eGFR ≥ 45: 0.64 (0.44–0.93) Diabetes: 0.64 (0.54–0.77) No Diabetes 0.82 (0.68–0.99) uACR < 30: 1.01 (0.66–1.55) uACR ≥ 30 to < 300: 0.91 (0.65–1.26) uACR ≥ 300: 0.67 (0.58–0.78)
SCORED [45] Sotagliflozin vs. placebo	*N* = 10,584; T2D with CKD (eGFR 25–60) and ≥ 1 CV risk factor	Composite of CV death or worsening HF events	Primary endpoint: 0.74 (0.63–0.88) HHF: 0.67 (0.55–0.82)	LVEF ≥ 50 and HF related criteria: 0.58 (0.38–0.90) uACR < 30 1.05 (0.76–1.46) uACR ≥ 30 to < 300 0.74 (0.57–0.97) uACR ≥ 300 0.60 (0.47–0.78)
CREDENCE [46] Canagliflozin vs. placebo	*N* = 4401; T2D with CKD (eGFR 30–90, uACR 300–5000); on RAAS blockade	Composite of ESKD, doubling of creatinine, or renal/CV death	Primary endpoint: 0.70 (0.59–0.82) Renal composite outcome: 0.66 (0.53–0.81) CV death: 0.78 (0.61-1.00)	eGFR < 45: 0.71 (0.56–0.89) eGFR ≥ 45 to < 60: 0.52 (0.38–0.72) eGFR ≥ 60: 0.81 (0.58–1.13) uACR < 1000 0.76 (0.55–1.04) uACR ≥ 1000 0.67 (0.55–0.81) BMI ≥ 30: 0.68 (0.54–0.86) Diabetes: 0.71 (0.57–0.88) No Diabetes: 0.68 (0.53–0.87) History of heart failure: 0.89 (0.61–1.31)

Effect of SGLT2i in Chronic Kidney Disease. Body mass index (BMI); Confidence interval (CI); Chronic kidney disease (CKD); Cardiovascular (CV); Estimated glomerular filtration rate (eGFR); Heart failure (HF); Hospitalization for heart failure (HHF) Hazard ratio (HR); Renin–angiotensin–aldosterone system (RAAS); Sodium-glucose cotransporter-2 inhibitor (SGLT2i); Type 2 diabetes mellitus (T2DM); Urinary albumin-to-creatinine ratio (uACR); Worsening renal function (WRF)

Subgroup analyses further indicated that the beneficial effects of SGLT2i on the primary composite outcome of reduction of kidney failure, cardiovascular death, and hospitalization for HF were evident in both patients with and without diabetes, with RRR ranging from 29% to 36% in patients with diabetes and from 18% to 50% in those without diabetes [43, 44, 46]. In addition, one study reported a 32% RRR in obese patients [46]. Collectively, these findings are consistent with the broader composite outcomes encompassing renal deterioration and cardiovascular events, including progression of kidney disease (e.g., eGFR < 10 mL/min/1.73 m², > 40% decline in eGFR, or kidney death), cardiovascular mortality, HF hospitalizations, and urgent HF visits.

Overall, SGLT2i demonstrated consistent reductions in the relative risk of heart failure hospitalization and exerted favourable effects on kidney outcomes across a broad range of metabolic comorbidities in patients with HFpEF and/or CKD.

### Effects of SGLT2i on pulmonary and skeletal muscle tissue

SGLT2i have been shown to significantly reduce pulmonary arterial pressure across multiple studies, highlighting a potential role in modulating pulmonary hemodynamic (Table 3). In clinical settings, SGLT2i attenuated exercise-induced post-capillary pulmonary hypertension–related hemodynamic abnormalities and exercise intolerance, supporting their potential as a therapeutic option in conditions characterized by exertional pulmonary pressure overload [47]. In patients with HFpEF, SGLT2i reduced pulmonary vascular load and improved right ventricle pulmonary artery coupling during exercise. These effects were accompanied by reductions in pulmonary capillary wedge pressure, indicating lower pulmonary circulatory pressures and translating into improvements in symptoms such as reduced dyspnoea [48]. 

**Table 3:** Effect of SGLT2i, GLP-1/GIP RA, and non-steroidal MRAs in HFpEF

Drug class	Heart	Kidney	Vasculature	Pulmonary	Skeletal muscle
SGLT2i	↓ fibrosis, ↓ inflammation, ↑ diastolic function	↓ hyperfiltration, ↓ fibrosis	↑ NO, ↓ ROS	↓ PAP, ↓ RV load	↑ mitochondrial function, ↑ insulin sensitivity
GLP-1/GIP RAs	↓ inflammation, ↑ Ca²⁺ handling	↓ albuminuria, ↓ inflammation	↓ inflammation, ↓ ROS, ↑ endothelial relaxation	↓ vascular inflammation	↓ ectopic fat,
nsMRA (Finerenone)	↓ hypertrophy, ↓ fibrosis	↓ CKD progression, ↓inflammation	↓ stiffness, ↓ inflammation	N/A	N/A

Effect of SGLT2i, GLP-1/GIP RA, and non-steroidal MRAs in HFpEF related organs. Calcium (Ca); Not applicable (N/A); Nitric oxide (NO); Pulmonary arterial pressure (PAP); Reactive oxygen species (ROS); Right ventricle (RV)

Beyond cardiopulmonary hemodynamic, SGLT2i also influence peripheral determinants of exercise tolerance, particularly skeletal muscle metabolism. In stable HFpEF patients with T2D, SGLT2i improved functional capacity [49], , although similar benefits were not observed in older, frail HFpEF populations [50]. Importantly, SGLT2i progressively reduced visceral adipose tissue in patients with type 2 diabetes and/or obesity without adversely affecting skeletal muscle mass or fat-free mass, indicating favourable alterations in body composition [51]. 

SGLT2i exert favourable effects on pulmonary hemodynamic, right ventricular remodelling, and skeletal muscle metabolism. The cardiopulmonary benefits appear to arise from combined structural and functional improvements within the pulmonary circulation and right heart, whereas skeletal muscle effects are predominantly metabolic, enhancing oxidative capacity and insulin sensitivity without directly increasing muscle strength.

### Mechanisms of action of SGLT2i and pleiotropic effects

#### Cardiac and renal mechanisms

SGLT2i exert multifaceted effects in HFpEF that extend well beyond glucose lowering. In the heart, they improve cardiac structure and function, including left ventricular dimensions, E/e′ ratio, and ejection fraction, and exert systemic hemodynamic effects such as reductions in body weight, plasma volume, and arterial stiffness, thereby lowering ventricular loading conditions and myocardial wall stress [52, 53]. In addition, SGLT2i reduce epicardial adipose tissue volume and modulate adipokine signalling, thereby dampening local and systemic inflammation and oxidative stress. This attenuation of adipose-driven inflammatory signalling contributes to protection against adverse myocardial remodelling [54]. 

In parallel, SGLT2i confer kidney protection in CKD through hemodynamic and non-hemodynamic mechanisms. They restore tubuloglomerular feedback and reduce intraglomerular pressure, thereby mitigating hyperfiltration and kidney injury [46, 55]. In parallel, SGLT2i attenuate kidney inflammation, oxidative stress, and fibrosis, partly through suppression of the AGE axis, leading to reduced mesangial expansion and interstitial fibrosis in both diabetic and non-diabetic CKD [56, 57]. Clinically, reductions in mean arterial pressure correlate with improvements in albumin-to-creatinine ratio, underscoring the close interplay between blood pressure lowering and kidney protection [58, 59]. Although glucosuria is consistently increased, natriuretic and diuretic effects may be attenuated under controlled sodium intake due to compensatory mechanisms such as activation of the RAAS, antidiuretic hormone release, and altered urea handling [60]. Importantly, the early modest decline in eGFR observed after initiation reflects a reversible hemodynamic adjustment rather than kidney injury and predicts long-term preservation of kidney function [43]. 

#### Pulmonary, skeletal muscle, and microvascular mechanisms

From a pulmonary perspective, experimental studies support the clinical benefits of SGLT2i. In preclinical models, SGLT2i attenuate pulmonary vascular remodelling, reduce right ventricular pressure overload, and improve right ventricular structure and function [61, 62]. 

When delving into skeletal muscle effects, SGLT2i appear to enhance muscle metabolic efficiency. In humans, SGLT2i increased mitochondrial oxidative capacity in prediabetic individuals [63], enhanced free fatty acid uptake [64], and improved insulin-stimulated skeletal muscle perfusion [65]. These findings indicate improved substrate utilization and microvascular delivery rather than direct anabolic effects.

Finally, SGLT2i exert direct vascular and endothelial effects, providing an additional mechanistic link to their cardio-renal benefits. SGLT2 expression in endothelial cells has been associated with low-grade inflammation, and its inhibition restores the balance between NO bioavailability and ROS, thereby reducing oxidative stress and vascular dysfunction [66]. In human cardiac microvascular endothelial cells, pro-inflammatory conditions impair contractility, which is restored by empagliflozin through improved NO bioavailability and reduced mitochondrial oxidative stress; similar protective effects are observed following exposure to uremic serum from patients with CKD [19, 67]. In clinical settings, SGLT2i enhance vasodilatory responses, including improved endothelium-independent vasodilation in T2D [68]. Notably, in patients with HFpEF, empagliflozin improved peripheral microvascular insulin-mediated vasodilation, suggesting enhanced insulin sensitivity and NO–mediated vasodilatory responses within the microcirculation [69]. 

SGLT2i exert integrated pulmonary, skeletal muscle, and microvascular effects that likely contribute to their clinical benefits. This coordinated improvement in cardiopulmonary coupling, peripheral metabolism, and endothelial function gives more insights in the extra-cardiac effects and symptom relief in HFpEF.

### GLP-1/GIP receptor agonists effects in CKMS

The clinical evidence supporting GLP-1 RAs is largely derived from cardiovascular outcome trials conducted in patients with T2D (Table 4). Most of these studies employed composite endpoints comprising non-fatal myocardial infarction, non-fatal stroke, and cardiovascular death, and consistently demonstrated RRR ranging from 9% to 27% for major adverse cardiovascular events, with only the ELIXA trial reporting a modest increase of 2% [70–77]. When focusing specifically on hospitalization for HF, the effects of GLP-1 RAs appear more modest and heterogeneous. Across trials, RRR of 4% to 21% were reported, with the notable exception of the SUSTAIN-6 trial, which did not demonstrate a benefit for this outcome [70, 72–78]. Importantly, hospitalization for heart failure was not stratified by LVEF ≥ 50% nor specifically analysed in HFpEF populations in these trials.

**Table 4:** Effect of GLP1RA in T2D. Including subgroup analysis of heart failure, obesity, and the prevalence of diabetes mellitus (if available)

Trial	Population	Primary endpoint	Primary outcomes HR (95%CI)	Subgroups HR (95%CI)
ELIXA [78] Lixisenatide vs. placebo	*N* = 6068; T2D with recent ACS	4-point MACE (CV death, MI, stroke, unstable angina)	Primary endpoint: 1.02 (0.89–1.17) HHF: 0.96 (0.75–1.23)	HR not reported; forest plot of subgroups
LEADER [70] Liraglutide vs. placebo	*N* = 9340; T2D	3-point MACE (CV death, MI, stroke)	Primary endpoint: 0.87 (0.78–0.97) HHF: 0.87 (0.73–1.05) Microvascular nephropathy events: 0.78 (0.67–0.92)	eGFR < 60: 0.69 (0.57–0.85) eGFR < 30: 0.89 (0.51–1.54) eGFR ≥ 30: 0.87 (0.77–0.97) BMI ≥ 30 0.82 (0.71–0.94) Chronic heart failure: 0.94 (0.72–1.21) Diabetes < 11 years: 0.82 (0.70–0.97) Diabetes ≥ 11 years: 0.90 (0.78–1.04)
SUSTAIN-6 [71] Semaglutide vs. placebo	*N* = 3297; T2D with CVD	3-point MACE (CV death, MI, stroke)	Primary endpoint: 0.74 (0.51–1.08) HHF: 1.11 (0.77–1.61) New/worsening nephropathy 0.64 (0.46–0.88)	eGFR < 30: 0.73 (0.27–1.97) eGFR < 60: 0.84 (0.48–0.92) BMI ≥ 30 0.84 (0.61–1.16) Chronic heart failure: 1.03 (0.64–1.66) Diabetes < 10 years: 0.82 (0.70–0.97) Diabetes > 10 years: 0.90 (0.78–1.04)
EXCSEL [72] Exenatide vs. placebo	*N* = 14,752; T2D	3-point MACE (CV death, MI, stroke)	Primary endpoint: 0.91 (0.83-1.00) HHF: 0.94 (0.78–1.13)	eGFR < 60 ml/min: 1.01 (0.86–1.19) eGFR renal function stage: I: 0.87 (0.71–1.06) II: 0.86 (0.75–0.99) III: 1.01 (0.86–0.99) IIIa: 0.97 (0.79–1.20) IIIb: 1.11 (0.84–1.47) IV: 1.26 (0.21–7.52) V: NA BMI ≥ 30: 0.89 (0.79-1.00)
AMPLITUDE-O [73] Efpeglenatide vs. placebo	*N* = 4076; T2D with CVD or CKD	3-point MACE (CV death, MI, stroke, including undetermined death)	Primary endpoint: 0.73 (0.58–0.92) HHF: 0.61 (0.38–0.98)	eGFR < 71.5 mg/ml 0.67 (0.50–0.91) BMI ≥ 31.9: 0.90 (0.64–1.26) Diabetes < 10 years: 0.79 (0.50–1.24) Diabetes ≥ 10 years: 0.70 (0.54–0.91)
HARMONY [74] Albiglutide vs. placebo	*N* = 9463; T2D with CVD	3-point MACE (CV death, MI, stroke)	Primary endpoint: 0.78 (0.68–0.90) CV death or HHF: 0.85 (0.70–1.04)	Heart failure: 0.70 (0.54–0.90) eGFR < 60: 0.93 (0.73–1.20) BMI ≥ 30 0.81 (0.68–0.97) Diabetes < 10 years: 0.67 (0.51–0.87) Diabetes ≥ 10 to < 20 years: 0.78 (0.63–0.97) Diabetes ≥ 20 years 0.90 (0.68–1.17)
REWIND [75] Dulaglutide vs. placebo	*N* = 9901; T2D (31.5% prior CVD)	3-point MACE (CV death, MI, stroke)	Primary endpoint: 0.88 (0.79–0.99) HHF: 0.93 (0.77–1.12) New/worsening renopathy: 0.85 (0.77–0.93)	BMI ≥ 30: 0.86 (0.74–0.98)
PIONEER 6 [76] Semaglutide vs. placebo	*N* = 3183; T2D with CVD or CKD	3-point MACE (CV death, MI, stroke)	Primary endpoint: 0.79 (0.57–1.11) HHF: 0.86 (0.48–1.55)	eGFR < 60 0.74 (0.41–1.33) BMI ≥ 30 0.95 (0.61–1.48)
SELECT [77] Semaglutide vs. placebo	*N* = 17,604; overweight/obesity with CVD (no diabetes)	3-point MACE (CV death, MI, stroke)	Primary endpoint: 0.80 (0.72–0.90) HHF: 0.79 (0.60–1.03) Nephropathy composite end point: 0.78 (0.63–0.96)	eGFR < 60: 0.69 (0.52–0.90) BMI ≥ 30 to < 35: 0.76 (0.64–0.91) BMI ≥ 35 to < 40: 0.93 (0.64–0.91) BMI ≥ 40 to < 45: 0.83 (0.55–1.26) BMI ≥ 45: 0.92 (0.51–1.65)

Effect of GLP1RA on Cardiac and Renal Endpoints. Body mass index (BMI); Confidence interval (CI); Chronic kidney disease (CKD); Cardiovascular (CV); Estimated glomerular filtration rate (eGFR); Glucagon-like peptide-1 receptor agonist (GLP1RA); Hospitalization for heart failure (HF); Hazard ratio (HR); Major Adverse Cardiovascular Event (MACE); Myocardial infarction (MI); Type 2 diabetes mellitus (T2D)

Beyond cardiovascular outcomes, GLP-1 RAs have shown favourable effects on microvascular complications. For kidney outcomes, including new or worsening nephropathy, RRR ranging from 13% to 36% have been reported, largely dependent on baseline kidney function as assessed by eGFR [70, 71, 73, 75, 77].

Subgroup analyses for the primary composite endpoint further suggest that the cardiovascular benefits of GLP-1 RAs vary across metabolic phenotypes. In individuals with obesity, RRR for primary cardiovascular outcomes ranged from 5% to 24%, depending on BMI [70–77]. Among patients with T2D, reductions in cardiovascular events ranged from 10% to 33%, with the magnitude of benefit influenced by disease duration [70, 71, 73, 74]. 

### GLP-1/GIP receptor agonists in HFpEF and CKD

Three notable trials investigated the efficacy of GLP-1/GIP RAs in patients with HFpEF or CKD (Table 5). The SUMMIT trial [6] and STEP-HFpEF trial [10], both conducted in patients with HFpEF and obesity, demonstrated clinically meaningful benefits. In the SUMMIT trial, treatment with the GLP-1/GIP RA tirzepatide resulted in a 38% RRR for the composite endpoint of cardiovascular death or worsening heart failure, accompanied by an improvement in quality of life. In the STEP-HFpEF trial, treatment with semaglutide was also associated with an improvement in quality of life, as well as a substantial reduction in body weight (− 10.7 kg) after 52 weeks (Table 4). When focusing specifically on hospitalization for HF, RRR of 44% in the SUMMIT trial and 92% in the STEP-HFpEF trial were observed.

**Table 5:** Effect of GLP1/GIP RA in HFpEF and CKD

Trial	Population	Primary endpoint	Primary outcome HR (95%CI)	Subgroups HR (95%CI)
SUMMIT [6] Tirzepatide vs. placebo	*N* = 731; HFpEF with BMI ≥ 30	Composite of CV death or worsening HF; Change in quality of life	Primary endpoint: 0.62 (0.41–0.95) Quality of life: Estimated difference 6.9 points (95%CI 3.3–10.6)	eGFR < 60: 0.61 (0.36–1.05) BMI < 35 0.47 (0.23–0.95) BMI ≥ 35 0.72 (0.43–1.23) Diabetes: 0.58 (0.31–1.07) No diabetes: 0.66 (0.37–1.18)
STEP-HFpEF [10] Semaglutide vs. placebo	*N* = 529; HFpEF with BMI ≥ 30	Change in quality of life; Change in body weight	Quality of life: Estimated difference 7.8 points (4.8–10.9) Body weight: Estimated difference, -10.7 (-11.9, -9.4)	Heart failure event: 0.08 (0.00-0.42)
FLOW [79] Semaglutide vs. placebo	*N* = 3533; T2D with CKD (eGFR 25–75, uACR ≥ 300 < 5000 mg/g); on RAAS blockade	Composite of the onset of kidney failure (dialysis, transplantation, eGFR of < 15 ml per minute per 1.73 m^2^) or ≥ 50% eGFR decline; kidney or CV related death	Primary endpoint: 0.76 (0.66–0.88)	Heart failure: 0.67 (0.49–0.93) eGFR ≥ 45 to < 60: 0.78 (0.58–1.04) eGFR ≥ 30 to < 45: 0.73 (0.59–0.92) eGFR < 30: 0.81 (0.58–1.13) uACR < 300: 0.86 (0.60–1.23) uACR ≥ 300: 0.74 (0.63–0.87) Diabetes < 15 years: 0.90 (0.72–1.13) Diabetes ≥ 15 years: 0.68 (0.56–0.82)

Effect of GLP1/GIP RA in HFpEF and CKD. Body mass index (BMI); Confidence interval (CI); Chronic kidney disease (CKD); Cardiovascular (CV); Estimated glomerular filtration rate (eGFR); Glucagon-like peptide-1 receptor agonist (GLP1RA); Heart failure (HF); Heart failure with preserved ejection fraction (HFpEF); Hazard ratio (HR); Quality of life (QoL); Renin–angiotensin–aldosterone system (RAAS); Type 2 diabetes mellitus (T2D); Urinary albumin-to-creatinine ratio (uACR)

Subgroup analyses of the primary composite outcome were performed in the SUMMIT trial and revealed consistent benefits across key clinical subgroups. RRR were consistent, ranging from 36% to 39% across eGFR categories and from 28% to 53% across body mass index categories (< 35 vs. ≥ 35 kg/m²). Additionally, the RRR was 34% in patients without diabetes and 42% in those with diabetes [6]. Although these trials enrolled fewer participants than earlier GLP-1 receptor agonist cardiovascular outcome trials in type 2 diabetes, they are among the first studies specifically designed to evaluate outcomes in patients with HFpEF.

Similarly, the FLOW trial [79], assessed kidney outcomes in patients with DKD. Treatment with semaglutide resulted in a 24% RRR for the composite kidney endpoint, defined as progression to dialysis or transplantation, an eGFR < 15 mL/min/1.73 m², a ≥ 50% decline in eGFR from baseline, or death from kidney-related or cardiovascular causes (Table 4). In patients with HF, a subgroup analysis demonstrated a RRR of 33%, while benefits across different eGFR categories ranged from 19% to 36%. Importantly, HF phenotype was not specified. The presence of diabetes further influenced treatment effects on the primary outcome, with RRR ranging from 10% to 32% depending on the cohort, whereas patients with elevated urine albumin-to-creatinine ratios experienced reductions of 14% to 26%.

Overall, GLP-1 RAs in HFpEF are associated with meaningful reductions in heart failure–related outcomes, particularly hospitalization, alongside consistent improvements in quality of life and body weight. These effects appear more pronounced than those observed in earlier cardiovascular outcome trials in patients with type 2 diabetes, although the smaller sample sizes of HFpEF-specific studies may limit generalizability. In parallel, evidence from CKD populations indicates that GLP-1RAs confer renal protection, supporting their potential to provide combined cardiovascular and kidney benefits.

### Mechanisms of action of GLP1RA in HFpEF

#### Cardiac and kidney mechanisms

GLP-1 receptors are expressed in human atrial tissue and within the cardiac conduction system; however, in HFpEF, the dominant therapeutic effects of GLP-1 RAs appear to be indirect, mediated primarily through systemic, vascular, and metabolic mechanisms rather than direct myocardial actions [80]. Upon receptor activation, GLP-1 RAs stimulate multiple intracellular signalling cascades, most notably the cyclic adenosine monophosphate–protein kinase A (cAMP/PKA) and PI3K–Akt pathways, leading to inhibition of NF-κB and subsequent reductions in inflammation, oxidative stress, and fibrotic signalling with concomitant improvements in endothelial function and microvascular perfusion [81, 82]. Although direct myocardial effects are not considered primary in HFpEF, emerging cellular data suggest that GLP-1 RAs may still exert cardiomyocyte-level benefits under pathological conditions. Human left ventricular cardiomyocytes exhibiting HFpEF-like phenotypes demonstrated improved contractility following GLP-1 RA treatment, mediated by reduced diastolic sarcoplasmic reticulum calcium leak and enhanced systolic calcium handling [83]. In parallel, GLP-1 RAs downregulate pro-inflammatory markers in epicardial adipose tissue, a metabolically active depot implicated in local myocardial inflammation and stiffness [83]. 

In obese HFpEF patients, GLP-1 RAs reduce body weight, circulatory volume, pressure overload, and systemic inflammation, thereby alleviating injury to the cardiovascular kidney axis and improving exercise capacity [84, 85]. Genetic studies corroborate this concept, demonstrating that GLP1 receptor expression is associated with modest reductions in kidney disease risk and progression independent of glycemia and body weight [86, 87]. Consistent with these findings, dual GLP-1/GIP RA in a kidney allograft fibrosis model reduces inflammatory cell infiltration, downregulates fibrotic pathways, enhances lipid metabolism, and improves structural and functional kidney outcomes [88]. 

GLP-1 RAs likely confer benefit in patients with HFpEF and CKD by reducing ventricular loading conditions via weight loss and natriuresis, improving endothelial and microvascular function, and attenuating inflammation and fibrosis in both myocardium and kidney.

#### Effects of GLP-1 receptor agonists on pulmonary and skeletal muscle tissue

In experimental models, GLP-1 RA attenuated pulmonary vascular remodelling and reduced right ventricular systolic pressure in mice. In human umbilical vein endothelial cells, GLP-1 RA also inhibited endothelial-to-mesenchymal transition and vascular inflammation [89]. Additional animal studies demonstrated pulmonary anti-inflammatory effects and improvements in vascular smooth muscle cell function [90]. Furthermore, GLP-1 RAs are associated with reductions in thigh muscle fat and adverse muscle composition, while preserving skeletal muscle mass, though without directly improving muscle strength [91, 92]. 

Beyond organ-specific effects, GLP-1 RAs exert systemic vascular and immunomodulatory actions. In patients with T2D, GLP-1 RA therapy was associated with reductions in circulating pro-inflammatory immune cell populations alongside increases in vascular regenerative progenitor cells, supporting a role in vascular repair and remodelling [93]. Consistent with these vascular effects, GLP-1 RAs improve endothelium-dependent relaxation and reduce vascular superoxide production in human subcutaneous resistance arteries [94]. 

GLP-1 RA primarily improve body composition by reducing ectopic fat and preserving muscle mass, while also enhancing microvascular function and metabolic flexibility. Their pulmonary effects appear to be mediated through anti-inflammatory and anti-remodelling pathways, which may explain the observed reduction in pulmonary hypertension risk. Unlike SGLT2i, GLP-1 RA show stronger effects on adiposity and inflammation, highlighting their complementary but distinct mechanisms of action.

### Mineralocorticoid receptor antagonists in HFpEF

The first major trial evaluating effects of MRA (Spironolactone) in patients with HFpEF was demonstrated in the TOPCAT trial, which showed a RRR of 17% in hospitalizations for HF but no significant improvement in the primary composite outcome (death from cardiovascular causes, aborted cardiac arrest, or hospitalization for heart failure) (Table 6) [95]. Subgroup analyses demonstrated a RRR of 20% in obese patients and in non-insulin treated diabetes a RRR of 10%. Further study analyses indicated that patients exhibiting a high-risk phenotype characterized by obesity, diabetes, CKD, and dyslipidaemia had the greatest incidence of the primary composite endpoint, with spironolactone failing to improve outcomes within this subgroup [96]. Despite these subgroup findings, MRAs have not achieved widespread adoption in the therapeutic management of HFpEF or its related comorbidities, also due to its side effects such as hyperkalaemia. Building upon these observations, recent attention has shifted toward non-steroidal MRAs, particularly finerenone, which exhibits distinct pharmacological and tissue-selective properties that may overcome some of the limitations associated with traditional steroidal MRAs such as spironolactone and eplerenone (Table 6).

**Table 6:** Effect of MRAs in HFpEF and CKD including subgroup analysis of obesity and the prevalence of diabetes mellitus (if available)

Trial	Population	Primary endpoint	Primary outcomes HR (95%CI)	Subgroups HR (95%CI)
TOPCAT [95] Spironolactone vs. placebo	*N* = 3445; HFmrEF/HFpEF	Composite of CV death, aborted cardiac arrest, or HF hospitalization	Primary endpoint: 0.89 (0.77–1.04) HHF: 0.83 (0.69–0.99)	eGFR < 60: 0.95 (0.77–1.17) BMI ≥ 31: 0.80 (0.66–0.98) No diabetes: 0.90 (0.73–1.11) Diabetes, insulin-treated: 0.80 (0.59–1.07) Diabetes, non-insulin treated: 0.90 (0.65–1.23)
FINEARTS-HF [7] Finerenone vs. placebo	*N* = 6016; HFmrEF/HFpEF	Composite of total worsening HF events or CV death	Primary endpoint: 0.86 (0.74–0.95) HHF: 0.82 (0.71–0.94)	eGFR < 60: 0.91 (0.78–1.07) uACR < 30: 0.81(0.67–0.97) uACR **≥** 30: 0.88 (0.74–1.05) BMI ≥ 30: 0.88 (0.74–1.05) Diabetes: 0.83 (0.69-1.00) No diabetes: 0.85 (0.71–1.01)
FIDELIO-DKD [97] Finerenone vs. placebo	*N* = 5734; T2D and CKD treated with RAAS and uACR 30 to < 300 and an eGFR 25–60 or uACR 300–5000 and eGFR 25 to < 75	Composite of kidney failure, ≥ 40% eGFR decline, or renal death	Primary endpoint: 0.82 (0.73–0.93) HHF: 0.86 (0.68–1.08) Worsening renal function: 0.81 (0.72–0.92) Kidney failure 0.87 (0.72–1.05)	eGFR < 25: 0.88 (0.48–1.64) eGFR ≥ 25 to < 45: 0.86 (0.73-1.00) eGFR ≥ 45 to < 60: 0.77 (0.61–0.96) uACR < 30: N/A uACR ≥ 30 < 300: 0.92 (0.49–1.72) uACR ≥ 300: 0.83 (0.73–0.93) BMI ≥ 30 0.98 (0.83–1.17)
FIGARO-DKD [98] Finerenone vs. placebo	*N* = 7437 T2D with CKD (eGFR 25–90; albuminuria) on RAAS blockade	4-point MACE (CV death, MI, stroke, HF hospitalization)	Primary endpoint: 0.87 (0.76–0.98) HHF: 0.71 (0.56–0.90)	eGFR < 25: 0.62 (0.10–3.83) eGFR ≥ 25 to < 45: 0.95 (0.73–1.25) eGFR ≥ 45 to < 60: 0.81 (0.61–1.06) uACR < 30: 0.67 (0.27–1.66) uACR ≥ 30 < 300: 0.87 (0.73–1.04) uACR ≥ 300: 0.90 (0.75–1.08) BMI ≥ 30 0.78 (0.66–0.92)
FIDELITY [99] Finerenone vs. placebo	*N* = 13,026; T2D with CKD (pooled FIDELIO + FIGARO)	4-point MACE Kidney: Kidney failure, ≥ 57% eGFR decline, or renal death	Primary outcome: 0.86 (95% CI 0.78–0.95) WRF: 0.77 (95% CI 0.67–0.88)	eGFR < 25: 0.48 (0.22–1.03) eGFR ≥ 25 to < 45: 0.94 (0.81–1.10) eGFR ≥ 45 to < 60: 0.80 (0.66–0.97) eGFR ≥ 60: 0.87 (0.74–1.01) uACR < 30: 0.59 (0.24–1.05) uACR 30-<300: 0.86 (0.73–1.02) uACR ≥ 300: 0.89 (0.79-<1.00) BMI ≥ 30 0.82 (0.73–0.93)

Effect of Finerenone in HFpEF and CKD. Body mass index (BMI); Confidence interval (CI); Chronic kidney disease (CKD); Cardiovascular (CV); Estimated glomerular filtration rate (eGFR); Heart failure (HF); Heart failure with preserved ejection fraction (HFpEF); Hazard ratio (HR); Hospitalization for heart failure (HHF); Major Adverse Cardiovascular Event (MACE); Renin–angiotensin–aldosterone system (RAAS); Type 2 diabetes mellitus (T2D); Urinary albumin-to-creatinine ratio (uACR)

In the FINEARTS-HF trial involving patients with HF with LVEF ≥ 40% [7], Finerenone reduced the composite of total worsening HF events and cardiovascular death with a RRR of 14%. Primarily driven by a risk reduction in hospitalization for HF of 18%. Benefits were consistent for the primary outcome across subgroups by kidney function with a relative risk reduction of 9% in those with eGFR < 60 mL/min/1.73m² and a more pronounced benefit of 29% in those with eGFR ≥ 60 mL/min/1.73m² (Table 5). Finerenone’ s effect on cardiovascular outcomes appeared consistent irrespective of albuminuria levels, BMI ≥ 30, and diabetes status assuming pleiotropic effects instead of only effects related to kidney function. In addition, subgroup analyses based on ejection fraction (< 60% vs ≥ 60%) showed no significant differences, indicating that these benefits extend to patients with HFpEF and are not limited to those with HFrEF.

The FIDELIO-DKD trial enrolled patients with T2D and CKD (varied albuminuria and eGFR stages) receiving RAAS blockade [97]. Finerenone reduced the composite kidney outcome (kidney failure, sustained ≥ 40% eGFR decline, or renal death) with a RRR of 18%. It decreased relative risk of worsening kidney function with 19%, 13% in reducing kidney failure, and 14% for HF hospitalizations. Kidney benefits were evident across eGFR subgroups, especially in 25 to < 60 mL/min/1.73 m² ranges. The effect varied less by albuminuria levels and BMI ≥ 30.The FIGARO-DKD trial focused on earlier CKD stages in T2D patients (Table 5) [98]. Finerenone reduced the composite cardiovascular outcome (cardiovascular death, nonfatal myocardial infarction or stroke, or HF hospitalization) by 13%, with pronounced reductions of 29% in HF hospitalizations. Cardiovascular benefits extended across eGFR strata and were notable in patients with BMI ≥ 30. Importantly to note that HF phenotype was not reported in these two trials.

The FIDELITY pooled analysis combined data from FIDELIO-DKD and FIGARO-DKD, confirming finerenone’ s RRR in the composite cardiovascular outcome with 14% and 23% for composite kidney outcome(Table 5) [99]. The kidney benefit was across eGFR strata, with the greatest benefit observed in patients with an eGFR of 45–60 mL/min/1.73 m² experiencing a RRR of 20%. Finerenone’ s effect on cardiovascular outcomes appeared consistent irrespective of albuminuria levels and was also seen in BMI ≥ 30.

Finerenone consistently improves cardiovascular and kidney outcomes in patients with CKD and T2D, with important benefits also observed in heart failure populations, including those with preserved ejection fraction. These benefits extend across a broad spectrum of kidney function, albuminuria levels, diabetes status, and obesity.

#### Mechanisms of action of MRA in HFpEF and CKD

Finerenone’s unique structure enables more balanced tissue distribution and minimizes off-target effects, thereby reducing the risk of hyperkalaemia [97, 98]. Modulating MR overactivation through aldosterone inhibition may also reduce oxidative stress and arterial stiffness [100], both of which are key contributors to cardiovascular complications in HFpEF patients. Preclinical studies further demonstrate that finerenone attenuates cardiac hypertrophy and fibrosis, limits vascular inflammation, and restores endothelial function [101, 102]. These mechanistic insights align with the clinical benefits observed in recent large trials including FINEARTS-HF, FIDELIO-DKD, FIGARO-DKD, and the FIDELITY pooled analysis in reducing adverse outcomes among patients with HFpEF, CKD, and T2D. However, it is important to note that these significant cardiovascular effects have not yet been systematically investigated in relation to pulmonary vascular remodelling or skeletal muscle adaptations, highlighting the need for further studies.

## Current guidelines

In this review, we have discussed that metabolic risk factors, such as obesity, T2D, and CKD are important contributors to HFpEF and are frequently accompanied by systemic and organ-specific microvascular dysfunction. While lifestyle interventions including a healthy diet, regular physical activity, and weight management remain fundamental, appropriate pharmacologic therapy tailored to each patient’s profile is also crucial.

Current guidelines emphasize a comprehensive, individualized approach to HFpEF management that accounts for these comorbidities. According to the 2022 AHA/ACC/HFSA [103] and 2023 ESC guidelines [2], SGLT2i are recommended for all patients with HFpEF to reduce HF hospitalizations and improve outcomes, irrespective of diabetes status. In contrast, steroidal MRAs have been recommended in the 2022 AHA/ACC/HFSA guideline (Class IIb recommendation) to reduce heart failure hospitalizations in patients with HFpEF. The combination of a SGLT2i and MRA demonstrated less discontinuation of MRAs and less hyperkalaemia in the SGLT2i users, making it an interesting interplay for the treatment of HFpEF [104]. GLP-1 RA are emerging as adjunct therapies, particularly in patients with obesity or T2D, promoting weight reduction, reduced insulin dependency due to enhanced glycaemic control, improved blood pressure regulation, and potential cardiovascular benefits [105]. The 2024 KDIGO CKD guidelines [106] and 2022 ADA/KDIGO consensus [107] similarly support SGLT2i and GLP-1 RA use in patients with CKD and/or T2D to prevent kidney disease progression and reduce cardiovascular events.

## Future perspectives

In HFpEF patients with CKD, obesity, and/or T2D, a personalized, mechanism-based approach may be preferable to routine upfront combination therapy. SGLT2i, GLP-1/GIP RA, and MRAs target overlapping but distinct pathways and should be selected based on dominant phenotype and symptom burden. Combined treatment with SGLT2i and GLP-1 RA has demonstrated additive cardiovascular and kidney benefits in patients with T2D [108], while adding an MRA may further reduce albuminuria and blood pressure [109]. However, robust evidence for dual or triple therapy in HFpEF remains limited, and no phase III trial has specifically evaluated GLP-1 RA in this population, despite promising data in patients with preserved LVEF.

Within CKMS-related HFpEF, therapeutic prioritization should be guided by individual patient characteristics rather than a uniform combination approach. In patients with microvascular dysfunction, SGLT2i are a rational first-line option given their beneficial effects on endothelial and microvascular function. In obesity and/or T2D, GLP-1/GIP RA are particularly advantageous due to their effects on weight reduction, metabolic control, and vascular health. In CKD, especially with albuminuria, the non-steroidal MRA finerenone has demonstrated kidney-protective effects and reduction in cardiovascular events. Integrating these therapies enables targeting of interconnected mechanisms, including congestion, metabolic dysregulation, inflammation, fibrosis, and renal stress, and may improve outcomes in this high-risk population. Future trials should evaluate combined use of SGLT2i, GLP-1/GIP RA, and MRA across the CKMS spectrum to define their synergistic potential.

Beyond current therapies, several novel compounds and combination strategies are under investigation. These include dual and triple incretin receptor agonists targeting GLP-1/GIP and glucagon receptors, as well as long-acting amylin analogues aimed at improving metabolic control while assessing cardiovascular and renal outcomes in CKD and DKD [110]. In parallel, aldosterone synthase inhibitors, such as vericostat, have shown promising renoprotective effects; in a phase II trial, vericostat combined with SGLT2i significantly reduced albuminuria, suggesting additive benefits beyond current MRA strategies [111]. Although not yet part of clinical practice, these therapies highlight opportunities to further individualize treatment by targeting residual metabolic and renal risk.

## Data Availability

No datasets were generated or analysed during the current study.

## Abbreviations

CKD: Chronic kidney disease
CKMS: Cardio-kidney metabolic syndrome
GLP-1/GIP RAs: Glucagon-like peptide-1/gastric inhibitory polypeptide receptor agonists
HF: Heart failure
HFpEF: Heart failure with preserved ejection fraction
MRA: Mineralocorticoid receptor antagonists
NADPH oxidase: Nicotinamide adenine dinucleotide phosphate oxidase
RAAS: Renin-angiotensin-aldosterone system
ROS: Reactive oxygen species
RRR: Relative risk reduction
SGLT2i: Sodium-glucose cotransporter 2 inhibitors
T2D: Type 2 diabetes mellitus
